# Sex-dependent predictors of binge drinking among males and females in North Dakota

**DOI:** 10.7717/peerj.20830

**Published:** 2026-02-24

**Authors:** Corey A. Day, Howard Onyuth, Grace Njau, Matthew Schmidt, Agricola Odoi

**Affiliations:** 1 Department of Biomedical and Diagnostic Sciences, College of Veterinary Medicine, University of Tennessee, Knoxville, TN, United States of America; 2 Division of Special Projects & Health Analytics, North Dakota Department of Health and Human Services, Bismarck, ND, United States of America

**Keywords:** Binge drinking, Mental distress, Predictors, Risk factors, North Dakota, United States, Logistic regression, Sex-differences

## Abstract

**Background:**

North Dakota has one of the highest rates of binge drinking in the United States, but little is known about how the predictors of binge drinking differ between males and females within the state. Therefore, the objective of this study was to identify and compare the sex-dependent predictors of binge drinking in North Dakota.

**Methods:**

Data were obtained from the North Dakota Behavioral Risk Factor Surveillance System (BRFSS) for the years 2017–2021. The BRFSS is a population based cross-sectional telephone survey administered annually by the North Dakota Department of Health and Human Services (NDDHHS) in coordination with the Centers for Disease Control and Prevention (CDC). The study population included all males and females aged ≥ 18 years in the North Dakota BRFSS database from 2017 to 2021 who responded to the questions “During the past 30 days, how many days per week or per month did you have at least one drink of any alcoholic beverage such as beer, wine, a malt beverage or liquor?” and “Considering all types of alcoholic beverages, how many times during the past 30 days did you have five or more drinks (for men) or four or more drinks (for women) on an occasion?.” Potential predictors of binge drinking were selected using a conceptual model and included: race, age, income, education, urban or rural residence, and frequent physical or mental distress. Separate binary logistic regression models of binge drinking were fitted for males and females.

**Results:**

The odds of binge drinking declined with increasing age in both sexes. Males who were Black or other races and ethnicities besides American Indian had lower odds of binge drinking than White males. Additionally, males had significantly higher odds of binge drinking if they lived in a rural county, had a household income ≥ $75,000, or had more than a high school education compared to those who lived in an urban county, had a household income < $75,000, or had no more than a high school education. White females had significantly higher odds of binge drinking than those who were Black, and females with frequent mental distress had significantly higher odds of binge drinking than females without frequent mental distress.

**Conclusions:**

The predictors of binge drinking differ between males and females in North Dakota. Geographic and socioeconomic factors were significant predictors in males, but not females, while frequent mental distress was only a significant predictor of binge drinking among females. Efforts to reduce binge drinking in this state should consider potential differences in programmatic needs between the sexes.

## Introduction

Excessive alcohol consumption is one of the most common causes of preventable deaths in the United States (US) (Centers for Disease Control Prevention, 2024c). Binge drinking, defined as consuming five or more drinks on one occasion for men and four or more drinks on one occasion for women, is the most common and expensive form of excessive drinking in the US, costing billions of dollars in healthcare spending, criminal justice costs, and lost productivity annually (Substance Abuse and Mental Health Services Administration, 2022). In 2020, among individuals 12 years and above, the prevalence of binge drinking in the past month was 24.9% in males compared to 19.7% in females (Substance Abuse and Mental Health Services Administration, 2022). In 2010, the combined costs of binge drinking in the US was $249 billion, accounting for 72% of lost labor and lower worker performance in the workplace, 17% of property damage, crashes, and criminal justice needs, and 11% of healthcare costs for injuries (Centers for Disease Control and Prevention, 2024a). On the state level, annual costs ranged from $488 million in North Dakota to $35 billion in California with the US government paying about $2 of every $5 spent to address the impacts of binge drinking (Substance Abuse and Mental Health Services Administration, 2022). However, approximately one in six people in the US engages in binge drinking weekly (Bohm et al., 2021), resulting in increased health risks such as injuries, violence, adverse pregnancy outcomes, and chronic diseases (Centers for Disease Control and Prevention, 2024b).

There are important sex and gender differences in the patterns and predictors of binge drinking (Shuey et al., 2025). Overall, males engage in excessive alcohol consumption three times more than women (Substance Abuse and Mental Health Services Administration, 2022). There are several biological and social factors that cause males to be more susceptible to excessive drinking, such as delayed brain development, higher levels of perceived alcohol use among peers, higher impulsivity, and societal gender roles (Levkovich, Yatzkar & Shenaar-Golan, 2025; Schulte, Ramo & Brown, 2009). However, the negative consequences of alcohol abuse may be more severe for females than males (Substance Abuse and Mental Health Services Administration, 2022), and the gap in excessive drinking rates between males and females is declining, indicating that binge drinking is becoming a more common problem among women (Kirsch et al., 2025; McKetta & Keyes, 2020). For example, compared to males, females are more susceptible to long-term health consequences such as alcohol induced liver inflammation and liver cancer, cardiovascular disease, memory blackouts, and future breast cancer (Centers for Disease Control and Prevention, 2024a; Centers for Disease Control and Prevention, 2024b). In addition, among young female adults, consumption of even one alcoholic drink is associated with an approximately 9% higher risk of developing breast cancer compared to abstainers (Substance Abuse and Mental Health Services Administration, 2023). Despite increasing alcohol use among females, few studies have conducted sex-specific investigations of binge drinking patterns, necessitating the need for further investigation (Bohm et al., 2021; Kirsch et al., 2025; Shuey et al., 2025).

Multiple studies have found that adults with a mental health condition have higher odds of engaging in excessive drinking behaviors than those without a mental health condition (Guckel et al., 2022; Puddephatt et al., 2021). One study in Finland found that frequent binge drinking was associated with poor mental health irrespective of socioeconomic factors, but in contrast, a study in southern Australia found that associations between excessive drinking and mental health conditions were rendered insignificant after controlling for sociodemographic factors (Guckel et al., 2022). In the US, there is evidence that women with mental distress have higher odds of consuming or abusing alcohol than those without mental distress (Hahm et al., 2023; Da Silva Júnior & De Monteiro, 2020). These have been attributed to women internalization of stress, anxiety, depression, and other mental health issues with binge drinking used as a coping mechanism (Levkovich, Yatzkar & Shenaar-Golan, 2025). Globally, mental health disorders and alcohol use disorders commonly coincide, as World Mental Health studies indicate that more than 40% of individuals with alcohol use disorders also have mental health disorders (Glantz et al., 2020).

Socioeconomic status (SES) plays a critical role in shaping patterns of binge drinking (Vargas-Martínez et al., 2020; World Health Organization, 2021; World Health Organization, 2024). Individuals with higher SES (income greater than $75,000) report drinking more frequently and in greater quantities than those with lower SES (income below $20,000) (World Health Organization, 2021). This has been partly attributed to greater disposable income, social environments that normalize binge drinking, and occupational cultures that increase both access to cheap alcohol and acceptability of binge drinking (World Health Organization, 2021; World Health Organization, 2024). In contrast, individuals with lower SES may engage in episodic binge drinking as a coping mechanism to chronic stress, financial insecurity, and limited access to mental health resources (Vargas-Martínez et al., 2020; World Health Organization, 2021). This may likely lead to a phenomenon known as the alcohol-harm paradox which refers to the observation that individuals with lower SES experience disproportionately greater alcohol-related harm than those with a higher SES even when consuming the same or lower amounts of alcohol (World Health Organization, 2021). In addition, it has been shown that higher education is associated with higher odds of individuals binge drinking at some point in their life, with those living in higher income neighborhoods binge drinking more than people in lower income neighborhoods (Substance Abuse and Mental Health Services Administration, 2022). At the community level, risk factors such as increased availability of alcohol through high alcohol outlet density, low alcohol prices especially when produced with local crops, widespread and targeted advertising and marketing to disadvantaged communities, weak regulatory environments that lower cost, and cultural and social norms that normalize heavy drinking worsen patterns of misuse (World Health Organization, 2021; World Health Organization, 2024). Thus, binge drinking impacts are most severe in disadvantaged communities where structural vulnerabilities amplify risks (Vargas-Martínez et al., 2020; World Health Organization, 2021).

North Dakota (ND), where one in ten residents currently living in poverty, is among the worst states for binge drinking ranking 3rd in the US with 26.81% of all adults (compared to 17% in the US), and 40.43% of ND young adults aged 18–25 years (compared to 29.73% in the US), including more than half (54.4%) of ND college students, reporting weekly binge drinking (Centers for Disease Control and Prevention, 2024a; Centers for Disease Control and Prevention, 2024b; North Dakota Department of Health and Human Services, 2025b). These high proportions which also include 12.4% of students experiencing blackouts and 6.8% physically injuring themselves, show a disproportionate burden and emphasize the need for targeted, state-specific studies and interventions (North Dakota Department of Health and Human Services, 2025b). In addition, ND ranks the highest in the US for the number of bars per capita at 49.73 bars per 100,000 people with a 2019 per capita consumption of ethanol from all alcoholic beverages at 3.19 gallons compared to the US at 2.38 gallons (National Institute on Alcohol Abuse and Alcoholism, 2021; North Dakota Department of Health and Human Services, 2025b). Moreso, 40.43% of ND young adults aged 18–25 years (compared to 29.73% in the US), and 54.4% of ND college students reported binge drinking one or more times per week, with 12.4% blacking out and 6.8% physically injuring themselves (North Dakota Department of Health and Human Services, 2025b). As of 2025 in ND, 16.7% of adult arrests were for driving under the influence of alcohol, 36% of fatal crashes and 23% of new domestic violence cases were alcohol related. Although, 76.3% of ND residents do not perceive binge drinking once or twice a week as risky (North Dakota Department of Health and Human Services, 2025b), there remains limited understanding of the sociodemographic predictors of this behavior within the state, or whether those predictors differ between males and females. These findings are critical for informing targeted health interventions, needs-based resource allocation, guiding future research, and aligns with the objectives of the Global Alcohol Action Plan 2022–2030 and Healthy People 2030 drug and alcohol goals (US Department of Health and Human Services, 2025; World Health Organization, 2024). Therefore, the objective of this study was to investigate sex-dependent predictors of binge drinking in North Dakota.

## Materials and Methods

### Ethics approval and consent to participate

This study was reviewed by the University of Tennessee Institutional Review Board (Number: UTK IRB-24-08175-XM) and determined to be eligible for exempt review under 45 CFR 46.101. Category 4ii: Secondary research for which consent is not required. Anonymized secondary data collected during the time period 2017–2021 were provided to researchers without identifiers. Investigators got access to the data for research purposes on April 23, 2024. Study subjects were not re-identified or contacted by the investigators. The study did not include minors since it only included individuals 18 years or older.

### Study area and data source

The study area and data sources have been described elsewhere by the investigators of the current study (Day et al., 2025). Specifically, this study was conducted in North Dakota, a state with 53 counties and a population of approximately 773,000 people (United States Census Bureau, 2022). The overall population is predominately non-Hispanic White (83%), followed by non-Hispanic American Indian (5%), Hispanic or Latino (4.8%), and non-Hispanic Black (3%). All other races or combinations of races comprise approximately 5% of the population (United States Census Bureau, 2022). Approximately 51% of the population is male and 49% is female. Twenty percent of males are ages 18–44 years, 12% are ages 45–64 years, and 7% are 65 years or older, while 18% of females are ages 18–44 years, 11% are 45–64 years, and 8% are 65 years or older.

Behavioral Risk Factor Surveillance System (BRFSS) data from the years 2017–2021 were obtained from the North Dakota Department of Health and Human Services (NDDHHS). Investigators got access to the data for research purposes on April 23, 2024. The BRFSS is a population based, cross sectional annual telephone survey of health-related behaviors, chronic health conditions, and usage of medical services administered annually by the NDDHHS in coordination with the Centers for Disease Control and Prevention (CDC). Eligible participants included non-institutionalized adults 18 years and older.

### Study population

The study population included all males and females aged ≥ 18 years in the North Dakota BRFSS database from 2017 to 2021 who responded to the questions “During the past 30 days, how many days per week or per month did you have at least one drink of any alcoholic beverage such as beer, wine, a malt beverage or liquor?” and “Considering all types of alcoholic beverages, how many times during the past 30 days did you have five or more drinks (for men) or four or more drinks (for women) on an occasion?”. The CDC used those questions to calculate a binary binge drinking variable. Respondents were categorized as binge drinkers if they reported that they drank alcohol in the last 30 days and reported that they had five or more drinks (males) or four or more drinks (females) on a single occasion. All other respondents were categorized as not binge drinkers.

### Selection of predictors

**Figure 1:**
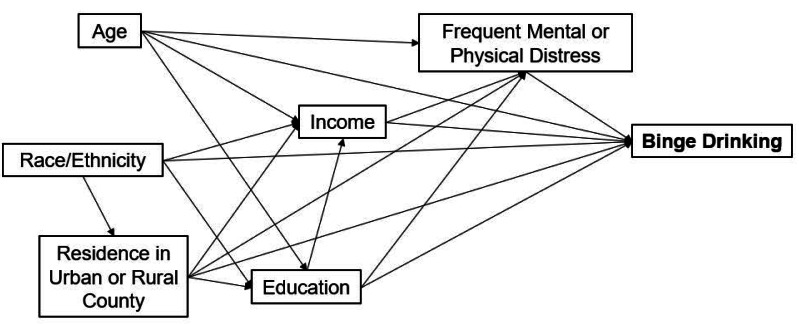
Conceptual causal model showing potential predictors of binge drinking among adults in North Dakota.

A conceptual causal model representing potential predictors of binge drinking was developed based on biological knowledge through a thorough literature review and the available questions asked in the BRFSS survey (Fig. 1). Potential predictors included age, race, residence in an urban or rural county, median household income, education level, frequent physical distress, and frequent mental distress. The outcome variable was binge drinking status for males and females.

### Data preparation

#### Re-coding of variables

All data cleaning and analysis were conducted using SAS Version 9.4 (SAS Institute Inc, 2013). Respondent age was categorized as 18–24 years, 25–34 years, 35–44 years, 45–64 years, and >65 years. Race and ethnicity were categorized as non-Hispanic White, non-Hispanic Black, non-Hispanic American Indian/Alaskan Native (AI), or Other Race/Ethnicity. Frequent mental distress was calculated based on respondents’ answer to the question “how many days during the past 30 days was your mental health not good?”, with 14 or more poor mental health days defining frequent mental distress (Centers for Disease Control Prevention, 2000). Household income was dichotomized as ≥ $75,000 or <$75,000 based on the median household income of North Dakota, which is approximately $75,000 (United States Census Bureau, 2022). Education was dichotomized as more than high school and high school or less using the CDC’s imputed education variable (_IMPEDUC). The binary _URBSTAT variable was used to define a county as urban or rural based on the 2013 National Center for Health Statistics urban-rural classification scheme (Centers for Disease Control Prevention, 2013).

#### Imputation

Missing data for the income variable were imputed because of a high non-response rate (16%). Data were imputed using the hot-deck method with weighted selection, in which donors with similar characteristics were chosen probabilistically, with replacement, according to respondent weights to preserve population representativeness and reduce bias (Andridge & Little, 2010; SAS Institute Inc, 2015). For example, assuming there are *m* recipient units and *r* donor units in an imputation cell, *ω*_*i*_ is the weight of the donor unit *i*, the ‘surveyimpute’ procedure of SAS (SAS Institute Inc, 2016a) selects, with replacement, a probability proportional to donor weight, *ω*_*i*,_ sample of size *m* from the *r* donors (SAS Institute Inc, 2015). The assumptions of this hot-deck method include: (a) missingness is random within imputation cells; (b) donor respondents are representative of non-respondents given auxiliary variables (education, age, race, binge drinking status, heavy drinking status, and county median household income); and (c) sampling weights appropriately adjust for selection probabilities to preserve the underlying population distribution (Andridge & Little, 2010; Shao & Tu, 2012). Imputation cells included respondents’ education, age, race, binge drinking status, heavy drinking status, and the median household income of the county where they resided based on the 2017–2021 5-year US Census Bureau American Community Survey (United States Census Bureau, 2022). Five donor cells were provided for each imputed response, and sampling weights for imputed values were divided by the number of donor cells. This ensured that the distribution of imputed values more closely reflects the underlying population distribution.

### Descriptive analyses

Descriptive analyses were conducted using the ‘surveyfreq’ procedure in SAS to account for the complex survey design of the BRFSS data (SAS Institute Inc, 2016a). The sampling weight (_LLCPWT), stratum (_STSTR), and cluster (_PSU) variables were specified in each analysis (Centers for Disease Control and Prevention, 2022). The original sampling weights provided by the CDC were divided by five to account for aggregation of five years of surveys (Centers for Disease Control and Prevention, 2022). Weighted frequencies and binge drinking prevalence proportions and their standard errors and 95% confidence levels for males and females were calculated separately. Domain analyses were used to ensure accurate calculation of standard errors and confidence intervals of weighted estimates (Lewis, 2013).

### Predictor investigation

Potential predictors from the conceptual model (Fig. 1) were assessed for bivariate (unadjusted) associations with binge drinking using binary logistic regression models for males and females, separately, by specifying respondent sex in the ‘domain’ argument of the ‘*surveylogistic*’ procedure in SAS software version 9.4 (Lewis, 2013; SAS Institute Inc, 2016b). Predictors whose bivariable associations had *p* < 0.2 were retained for further analysis. In this logistic regression, the outcome (binge drinking status) was dichotomous. The assumptions of logistic regression include: (a) observations are independent from each other, and (b) continuous predictors have linear relationships with the outcome (Dohoo, Martin & Stryhn, 2012). The selected predictors from the bivariable analysis were then assessed for potential collinearity issues using pairwise Spearman’s correlation tests. A conservative threshold of (r_s_ < —0.70—) was used to identify pairs of highly correlated variables, but none were highly correlated. To investigate the associations of predictors with the outcome after adjusting for covariates, separate multivariable logistic models were then fitted containing all potential predictors for males and females. Variables were included in the models based on: (a) the conceptual model developed based on evidence from a thorough literature review and theoretical relevance to binge drinking; and (b) statistical consideration from the bivariable analyses and pairwise spearman’s correlation tests. Effect estimates were reported as odds ratios with 95% confidence intervals, and the predictors were considered significant if their *p*-value was less or equal to 0.05. All models accounted for complex survey designs by specifying the sampling weight, strata, and cluster variables using the ‘surveylogistic’ procedure in SAS software version 9.4 (SAS Institute Inc, 2016b). The predictive abilities of the final models were assessed using a receiver operating curve (ROC) and area under the curve (AUC) (Agnelli, 2014).

## Results

### Descriptive analysis

The weighted totals of males and females who responded to questions related to binge drinking were 283,526 and 273,127 persons, respectively. Most of the population was non-Hispanic White (87%), 4% were non-Hispanic American Indian, 3% were non-Hispanic Black, and 6% were other races or ethnicities. Most males (71%) and females (84%) lived in counties designated as urban. After imputation of missing income data, most males (57%) and females (62%) had household incomes <$75,000, while 63% of males and 69% of females had more than a high school education.

The prevalence of binge drinking was 28% among males and 16% among females. Binge drinking prevalence declined with increasing age in both sexes (Table 1). The Black population had the lowest binge drinking prevalence proportions among both males and females. The confidence levels of binge drinking prevalence proportions overlapped among all other races or ethnicities in both sexes indicating no significant differences. Binge drinking was more common among males and females with household incomes ≥ $75,000 compared to those with incomes <$75,000. Similarly, binge drinking was also more common among males and females with more than a high school education than those with no more than a high school education, although the 95% confidence intervals between the categories overlapped for females indicating no statistically significant differences. Both males and females with frequent mental distress reported higher prevalence proportions of binge drinking than those without frequent mental distress. Males without frequent physical distress had a higher prevalence of binge drinking than males with frequent physical distress, but there was no substantial difference in binge drinking prevalence between females with and without frequent physical distress (Table 1).

**Table 1:** Population characteristics and binge drinking prevalence of males and females in North Dakota, 2017—2021.

	Male		Female	
	(Binge drinking prevalence = 28%)		(Binge drinking prevalence = 16%)	
Characteristic	Total (SE^a^)	% Binge drinking (CI^b^)	Total (SE^a^)	% Binge drinking (CI^b^)
**Age**				
18–24	41,703 (1,575)	39 (36, 43)	37,341 (1,828)	26 (22, 30)
25–34	57,461 (1,695)	39 (36, 42)	48,569 (1,771)	24 (21, 27)
35–44	46,174 (1,383)	33 (30, 35)	42,645 (1,412)	21 (18, 24)
45–64	81,928 (1,436)	22 (21, 24)	79,455 (1,342)	14 (13, 15)
65 or older	51,160 (896)	9 (8, 10)	65,117 (906)	3 (3, 4)
**Race**				
White	240,921 (2,445)	28 (27, 29)	236,439 (2,652)	16 (15, 17)
Black	8,190 (716)	15 (9, 21)	6,306 (774)	5 (2, 9)
American Indian	10,988 (678)	30 (24, 36)	11,916 (743)	17 (12, 21)
Other	20,107 (1,118)	24 (19, 29)	17,140 (1,142)	19 (13, 24)
**Urban/Rural**				
Urban	210,637 (2,572)	28 (27, 29)	202,946 (2,845)	15 (15, 17)
Rural	72,724 (1,313)	26 (25, 28)	70,041 (1,259)	15 (14, 17)
**Median household income**				
<$75,000	160,394 (2,238)	25 (24, 27)	169,770 (2,475)	15 (13, 16)
≥ $75,000	121,501 (1,926)	30 (29, 32)	102,450 (1,891)	18 (16, 20)
**Education**				
High school or less	105,876 (2,018)	25 (23, 27)	84,466 (1,984)	14 (13, 16)
More than high school	177,650 (2,225)	29 (28, 30)	188,875 (2,245)	17 (15, 18)
**Frequent Mental Distress**				
<14 days	256,351 (2,588)	27 (26, 28)	232,775 (2,720)	15 (13, 15)
≥ 14 days	24,898 (1,088)	33 (28, 37)	37,192 (1,446)	25 (22, 29)
**Frequent Physical Distress**				
<14 days	258,104 (2,641)	28 (27, 29)	241,012 (2,862)	16 (15, 17)
≥ 14 days	22,879 (889)	20 (17, 24)	28,836 (1,052)	15 (12, 19)

**Notes.**

a Standard error.

b 95% Confidence Interval.

### Predictors of binge drinking

Univariable associations of each potential predictor with binge drinking among males and females are presented in Table 2. Based on a significance threshold of *p* < 0.05, age, race, income, education, and frequent mental distress had significant bivariate associations with binge drinking in both sexes. Frequent physical distress had a significant bivariate association with binge drinking in males (Odds Ratio (OR) = 0.7; 95% CI [0.5–0.8]; *p* = 0.001) but not females (OR = 0.9; 95% CI [0.7–1.3]; *p* = 0.7). Residence in a rural or urban county was not significantly associated with binge drinking in the univariable model for males (OR = 0.9; 95% CI [0.8–1.0]; *p* = 0.05), and females (OR = 0.9; 95% CI [0.8–1.1]; *p* = 0.1).

**Table 2:** Results of univariable logistic regression models of binge drinking for males and females in North Dakota, 2017–2021 with unadjusted odds ratios and *p*-values.

	Male		Female	
Characteristic	Odds ratio (CI^a^)	*p*-value	Odds ratio (CI^a^)	*p*-value
**Age**		**<0.001**		**<0.001**
25–34	1.0 (0.8, 1.2)	0.9	0.8 (0.6, 1.0)	0.5
35–44	0.7 (0.6, 0.9)	0.004	0.8 (0.6, 1.0)	0.07
45–64	0.5 (0.4, 0.5)	<0.001	0.5 (0.4, 0.6)	<0.001
65 or older	0.2 (0.1, 0.2)	<0.001	0.1 (0.08, 0.1)	<0.001
18–24 (reference)				
**Race**		**0.003**		**0.03**
Black	0.5 (0.3, 0.7)	0.001	0.3 (0.1, 0.7)	0.006
American Indian	1.1 (0.8, 1.4)	0.5	1.1 (0.8, 1.5)	0.7
Other	0.8 (0.5, 1.0)	0.09	1.20 (0.8, 1.7)	0.3
White (references)				
**Urban/Rural**		**0.05**		**0.1**
Rural	0.9 (0.8, 1.0)	0.2	0.9 (0.8, 1.1)	0.3
Urban				
**Median household income**		**<0.001**		**<0.001**
<$75,000	0.8 (0.7, 0.9)	<0.001	0.8 (0.7, 0.9)	0.001
≥ $75,000 (reference)				
**Education**		**<0.001**		**<0.001**
High school or less	0.8 (0.7, 0.9)	<0.001	0.8 (0.7, 1.0)	0.05
More than high school (reference)				
**Frequent mental distress**		**<0.001**		**<0.001**
≥ 14 days	1.3 (1.1, 1.6)	0.01	2.0 (1.6, 2.5)	<0.001
<14 days (reference)				
**Frequent physical distress**		**<0.001**		0.4
≥ 14 days	0.7 (0.5, 0.8)	0.001	0.9 (0.7, 1.3)	0.7
<14 days (reference)				

**Notes.**

a 95% Confidence Interval.

The *p*-values in bold indicate statistically significant associations based on a critical alpha of 0.2.

The results of the multivariable models of binge drinking in males and females are presented in Table 3. In the multivariable model for males, age, race, residence in an urban or rural county, income, and education were significant predictors of binge drinking, but frequent mental distress and frequent physical distress were not. The odds of binge drinking were significantly lower among males older than 34 years (35–44 years (OR = 0.7; 95% CI [0.5–0.8]; *p* < 0.001), 45–64 years (OR = 0.4; 95% CI [0.3–0.5]; *p* < 0.001), 65 years and older (OR = 0.1; 95% CI [0.1–0.2]; *p* < 0.001)) compared to those who were ages 18–24 years. However, there was no significant difference in the odds of binge drinking between males aged 18–24 years and males aged 25–34 years (OR = 0.9; 95% CI [0.8–1.1]; *p* = 0.5). Males who were non-Hispanic Black (OR = 0.3; 95% CI [0.2–0.5]; *p* < 0.001) or other races/ethnicities (OR = 0.6; 95% CI [0.4–0.8]; *p* < 0.001) besides American Indian (OR = 0.9; 95% CI [0.7–1.2]; *p* = 0.7) had significantly lower odds of binge drinking than non-Hispanic White males. Additionally, males had significantly higher odds of binge drinking if they lived in a rural county (OR = 1.1; 95% CI [1.0–1.3]; *p* = 0.04), had a household income ≥$75,000 (OR = 1.2; 95% CI [1.04–1.3]; *p* = 0.008), or had more than a high school education (OR = 1.3; 95% CI [1.1–1.4]; *p* < 0.001) compared to males who lived in urban counties, had a household income <$75,000, or had no more than a high school education, respectively. The area under the curve (AUC) of the multivariable model of binge drinking for males was 0.70.

**Table 3:** Results of multivariable logistic regression models results showing predictors of binge drinking among males and females in North Dakota, 2017–2021 with adjusted odds ratios and *p*-values.

	Male		Female	
Characteristic	Odds ratio (CI^a^)	*p*-value	Odds ratio (CI^a^)	*p*-value
**Age**		**<0.001**		**<0.001**
25–34	0.9 (0.8, 1.1)	0.5	0.8 (0.6, 1.1)	0.2
35–44	0.7 (0.5, 0.8)	**<0.001**	0.6 (0.5, 0.9)	**0.003**
45–64	0.4 (0.3, 0.5)	**<0.001**	0.4 (0.3, 0.5)	**<0.001**
65 or older	0.1 (0.1, 0.2)	**<0.001**	0.1 (0.07, 0.1)	**<0.001**
18–24 (reference)				**<0.001**
**Race**		**<0.001**		**0.001**
Black	0.3 (0.2, 0.5)	**<0.001**	0.2 (0.1, 0.4)	**0.002**
American Indian	0.9 (0.7, 1.2)	0.7	0.8 (0.6, 1.2)	0.3
Other	0.6 (0.4, 0.8)	**<0.001**	0.7 (0.5, 1.1)	0.1
White (references)				
**Urban/Rural**				
Rural	1.1 (1.0, 1.3)	**0.04**	1.1 (0.9, 1.3)	0.1
Urban				
**Median household income**				
≥ $75,000	1.2 (1.04, 1.3)	**0.008**	1.1 (0.95, 1.3)	0.2
<$75,000 (reference)				
**Education**				
More than high school	1.3 (1.1, 1.4)	**<0.001**	1.2 (0.96, 1.4)	0.1
High school or less (reference)				
**Frequent mental distress**				
≥ 14 days	1.2 (0.9, 1.4)	0.2	1.6 (1.3, 2.0)	**<0.001**
<14 days (reference)				
**Frequent physical distress**				
≥ 14 days	0.8 (0.7, 1.1)	0.3	1.0 (0.7, 1.3)	0.9
<14 days (reference)				

**Notes.**

a 95% Confidence Interval.

The *p*-values in bold indicate statistically significant associations based on a critical alpha of 0.05.

In the multivariable model of binge drinking for females, only race, age, and frequent mental distress had significant associations with binge drinking after adjusting for covariates, while residence in an urban or rural county, income, education, or frequent physical distress did not have significant associations with the outcome. Similar to the results for males, the odds of binge drinking among females were significantly lower among those aged 35 years or older (35–44 years (OR = 0.6; 95% CI [0.5–0.9]; *p* = 0.003), 45–64 years (OR = 0.4; 95% CI [0.3–0.5]; *p* < 0.001), 65 years and above (OR = 0.1; 95% CI [0.07–0.1]; *p* < 0.001)) compared to those who were ages 18–24 years, but there was no significant difference between females aged 18–24 years or those aged 25–34 years (OR = 0.8; 95% CI [0.6–1.1]; *p* = 0.2). Females who were non-Hispanic Black (OR = 0.2; 95% CI [0.1–0.4]; *p* = 0.002) had lower odds of binge drinking than non-Hispanic White females, but there were no significant differences in binge drinking between other races and ethnicities (OR = 0.7; 95% CI [0.5–1.1]; *p* = 0.1) and non-Hispanic White females. The odds of binge drinking were also higher among females with frequent mental distress (OR = 1.6; 95% CI [1.3–2.0]; *p* < 0.001) than those who did not have frequent mental distress. The AUC of the multivariable model of binge drinking for females was 0.74.

## Discussion

This study identified predictors of binge drinking among men and women in North Dakota using data from the 2017–2021 BRFSS. The results suggest that the significant predictors of binge drinking differ between males and females after adjusting for covariates. Although race and age were significant predictors of binge drinking in both sexes, socioeconomic metrics (*i.e.,* education and income) and residence in an urban or rural county only maintained significant associations in the model of binge drinking among males, while frequent mental distress only maintained a significant association in the model of binge drinking among females. These results add to evidence of sex and gender differences in binge drinking patterns between males and females (Wilsnack et al., 2018) and are useful for the NDDHHS to develop programs that consider differences in binge drinking between the sexes.

In this study, the finding that both income and education levels had significant positive associations with the odds of binge drinking only among males (and not females) after controlling for covariates, may reflect gendered social norms and cultural expectations surrounding alcohol use as reported by previous studies (McKetta et al., 2021; McKetta & Keyes, 2020; Platt et al., 2021; Shuey et al., 2025). For example, males with higher prestige and higher income occupations may have greater exposure to professional and social environments where binge drinking is normalized, and socioeconomic resources provide greater access to alcohol (McKetta et al., 2021). On the contrary, higher education in females has been associated with increased health awareness and greater social stigma against binge or heavy drinking (McKetta et al., 2021; Shuey et al., 2025). National studies indicate that women’s binge drinking is increasing disproportionately in higher prestige occupations, likely driven by shifting gender roles and occupational norms, targeted social media campaigns and marketing towards females, and designing of alcohol bottles to appear appealing based on tastes and preferences of females, especially those with higher SES (McKetta et al., 2021; Platt et al., 2021). However, there is also evidence that the frequency of binge drinking is higher among binge drinkers with incomes <$25,000 than among binge drinkers with incomes >$75,000 (Substance Abuse and Mental Health Services Administration, 2022). Notably, other forms of excessive alcohol use, such as heavy drinking (*e.g.*, 15 or more drinks per week for men or eight or more drinks per week for women), are more common among individuals of lower socioeconomic status than people of higher socioeconomic status (Cerdá, Johnson-Lawrence & Galea, 2011). As of 2025, evidence-based adult binge drinking prevention strategies used in North Dakota include: (1) the Responsible Beverage Service Training, designed to educate owners, managers, servers, and sellers at alcohol establishments about strategies to avoid illegally selling alcohol to intoxicated customers; (2) increasing price of alcohol through municipal, state, or federal legislation that raises the excise tax on alcohol; (3) restrictions on alcohol discount promotions such as happy hours, free beverages (*e.g.*, ‘Ladies drink free’), additional servings, reduced price (*e.g.*, 2 for 1), unlimited beverages (*e.g.*, $10 all you can drink); (4) alcohol advertising restrictions and limits in public places, restricting alcohol sponsorship of events and other promotions, and counter marketing and counter advertising campaigns/alcohol warning posters; (5) Screening, Brief Intervention, and Referral to Treatment, a practice used to identify, reduce, and prevent problematic use, abuse, and dependence on alcohol in medical settings, and workplaces; and (6) media advocacy to dispel misperceptions, gain community support and enhance prevention efforts (North Dakota Department of Health and Human Services, 2025a). Based on the results of this study, the above prevention efforts/programs should target higher-income, higher educated males.

The observed association between frequent mental distress and binge drinking for females may be associated with the use of alcohol as a coping mechanism for stress, anxiety, or depressive symptoms compounded by gender specific social and cultural pressures that influence drinking behavior and the perception of alcohol as a normative stress-relief strategy (Kirsch et al., 2025; McKetta et al., 2021; Substance Abuse and Mental Health Services Administration, 2023). Mental health disorders including depression and anxiety are known to be associated with excessive alcohol use (O’Hare, 1995; Jane-Llopis & Matytsina, 2006; Glantz et al., 2020). One study of undergraduate students found that females who consume alcohol experience anxiety more often than men who consume alcohol (Torres et al., 2023), and a cross-sectional study in Korea from 2010 to 2015 found that women with alcohol use disorders experienced a stronger association with negative psychological outcomes than men (Jeong et al., 2019). It is possible that self-perceptions of poor mental health days differ between males and females, such that males may be less likely to report frequent mental distress than females even when suffering from a similar mental health burden. An important note is that the observed association between frequent mental distress and binge drinking does not necessarily imply a causal relationship, and it remains unclear whether these factors influence each other or are jointly affected by other underlying variables. Suffice it to say that additional research on these associations and on the sex-dependent relationships between alcohol use and self-perceived mental health are warranted.

The observed higher prevalence of excessive alcohol use in males than females (Bohm et al., 2021; Platt et al., 2021; Substance Abuse and Mental Health Services Administration, 2022) may be due to: (a) the fact that males not to perceive the risks of excessive alcohol consumption as highly as females do, and (b) males may be more susceptible than females to social pressures, peer influence, impulsivity, aggression, and conduct-related problems that make them engage in excessive alcohol consumption, (Schulte, Ramo & Brown, 2009). Females are more likely than males to engage in rumination (repetitive focus on negative emotional experiences) and therefore, they tend to internalize problems such as stress, depression, and anxiety. This may increase their vulnerability to using alcohol as a coping mechanism and therefore elevate their risk of binge drinking (Levkovich, Yatzkar & Shenaar-Golan, 2025).

The observed odds of binge drinking that were significantly lower among males and females aged 35 years and older compared to those aged 18–24 years, with no significant differences observed between individuals aged 18–24 years and those aged 25–34 years, may be due to binge drinking varying across the lifespan, with different drivers being responsible for this behavior among adolescents, young adults, and older adults (Haass-Koffler et al., 2020; Han et al., 2019; Krieger et al., 2018; Patrick et al., 2024). These patterns show the prevalence of excessive drinking and binge drinking peak during young adulthood and decline steadily thereafter (Substance Abuse and Mental Health Services Administration, 2021). Developmental, biological, and social factors likely account for these age differences. During adolescence, early initiation of drinking, peer and family influences, a history of trauma/adverse childhood experiences, and untreated psychiatric conditions (*e.g.*, major depressive disorders, anxiety, bipolar disorder, schizophrenia, *etc.*) are major risk factors (Patrick et al., 2024; Substance Abuse and Mental Health Services Administration, 2021). In young adulthood (18–25 years), increased independence, reduced parental oversight, heightened exposure to peer influence, other substance use, drinking to cope, social norms and environments that encourage heavy drinking, college/university campuses, bars, and parties, contribute to elevated binge drinking (Hahm et al., 2023; Krieger et al., 2018; Platt et al., 2021). When individuals transition to full adulthood, stable employment, marriage, and parenthood binge drinking declines (McKetta et al., 2021; Platt et al., 2021). Risk factors among individuals 65 years and older shift towards psychosocial stressors such as retirement, bereavement, or social isolation, along with comorbid psychiatric conditions, long term drinking habits, and physiological changes that may lead individuals to binge drinking (Eastman, Finlay & Kobayashi, 2021; Substance Abuse and Mental Health Services Administration, 2020). However, older adults are less likely to binge drink, partly due to physiological changes that reduce alcohol tolerance and comorbidities that discourage alcohol consumption (Substance Abuse and Mental Health Services Administration, 2020). While overall binge drinking prevalence is lower in this group (10.7%), older adults who binge drink were likely to be male, had a higher prevalence of current tobacco and or cannabis use, and a lower prevalence of two or more chronic diseases compared to those who did not binge drink (Han et al., 2019). Importantly, due to increased alcohol sensitivity, binge drinking in this population may occur at lower levels of alcohol consumption (Han et al., 2019; Substance Abuse and Mental Health Services Administration, 2020).

It should be noted that older adults are more likely than adolescents or younger adults to experience the negative effects of binge drinking due to age related physiological changes and an increased risk of alcohol-drug interactions (Substance Abuse and Mental Health Services Administration, 2020). Therefore, screening and treatment approaches for binge drinking in this age group should be tailored to the unique physical, emotional, and social needs of older adults, with senior friendly tools (Jeitani et al., 2024; Substance Abuse and Mental Health Services Administration, 2020).

### Study strengths and limitations

This is the first study to use BRFSS data to identify and compare predictors of binge drinking among men and women in North Dakota, providing an improved understanding of the sex-dependent risk factors in this population. One of the novel aspects of the study was the investigation of patterns and risk factors among males and females as separate groups, which allowed for the identification of unique associations between predictors and the outcome between the sexes. These findings are useful for the North Dakota Department of Health and Human Services to guide the implementation of programs to reduce the health impacts and societal costs of binge drinking in these populations.

The use of BRFSS data in this study means that drinking behaviors were based on self-reporting and not direct measurements, which may introduce selection, recall and desirability biases which may potentially result in biased estimates of the prevalence of binge drinking and predictor variables. This limitation included the definition of binge drinking, which was based on the CDC definition that only considers the number of drinks consumed on a single occasion. The National Institute on Alcohol Abuse and Alcoholism defines binge drinking as consumption which leads to a blood alcohol concentration to ≥ 8.0 grams of alcohol per deciliter of blood, a value that is expected to be reached by a typical adult male after five drinks or a typical adult female after four drinks consumed within 2 h (National Institute on Alcohol Abuse and Alcoholism, 2023). The influence of alcohol consumption on blood alcohol concentration varies based on several factors including body weight. Therefore, measuring binge drinking by the number of drinks may provide a biased estimate. Despite this limitation, the estimates derived from BRFSS data are reliable and valid as they correspond well with findings from surveys based on face-to-face interviews including the National Health Interview Survey (NHIS), and the National Health and Nutrition Examination Survey (NHANES).

Although BRFSS is a large, population-based survey, certain groups may be under-represented, including individuals without reliable telephone access, those with unstable housing, or individuals less likely to participate in health surveys, which may affect sample representativeness. As with observational studies, unmeasured confounders such as co-occurring substance use, access to healthcare, physical health status, and social support may influence both binge drinking and frequent mental distress, but these may not be fully accounted for in this study.

The BRFSS is a population-based, cross-sectional telephone survey and thus provides a snapshot of health behaviors and health outcomes at a single point in time. Therefore, analyses using BRFSS data can identify associations between variables (*i.e.,* frequent mental distress and binge drinking) but cannot be used to infer causal relationships. Future studies using longitudinal designs are needed to assess and establish causality.

## Conclusions

The significant predictors of binge drinking differ between men and women of North Dakota. Further research should be conducted to investigate why the effect of mental distress differed in magnitude between the sexes, and to better understand whether frequent mental distress causes binge drinking or vice versa. Public health officials who aim to reduce binge drinking in North Dakota should consider that program needs may differ by sex.

##  Supplemental Information

10.7717/peerj.20830/supp-1 Supplemental Information 1 Data All variables used in the investigation.

10.7717/peerj.20830/supp-2 Supplemental Information 2 Codebook

## Additional Information and Declarations

### Data Availability

The following information was supplied regarding data availability: The raw data and code are available in the Supplemental Files.
